# (*E*)-*N*′-(3,5-Dibromo-2-hydroxy­benzyl­idene)-2-methoxy­benzohydrazide

**DOI:** 10.1107/S1600536809022168

**Published:** 2009-06-17

**Authors:** Guo-Biao Cao, Xu-Hui Lu

**Affiliations:** a Department of Chemistry, Ankang University, Ankang Shanxi 725000, People’s Republic of China

## Abstract

The title compound, C_15_H_12_Br_2_N_2_O_3_, was synthesized by the reaction of 3,5-dibromo-2-hydroxy­benzaldehyde with an equimolar quantity of 2-methoxy­benzohydrazide in methanol. The dihedral angle between the two benzene rings is 3.4 (2)° and intra­molecular O—H⋯N and N—H⋯O hydrogen bonds are observed in the mol­ecule. The crystal studied was an inversion twin with a 0.513 (19):0.487 (19) domain ratio.

## Related literature

For related structures, see: Mohd Lair *et al.* (2009 ▶); Fun *et al.* (2008 ▶); Li & Ban (2009 ▶); Zhu *et al.* (2009 ▶); Yang (2007 ▶); You *et al.* (2008 ▶). For our previous work in this area, see: Qu *et al.* (2008 ▶); Yang *et al.* (2008 ▶). For reference structural data, see: Allen *et al.* (1987 ▶).
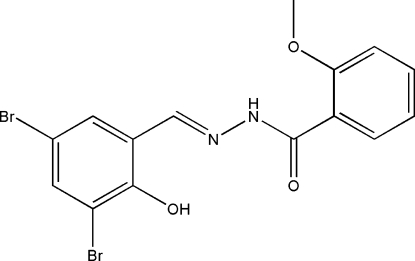


## Experimental

### 

#### Crystal data

C_15_H_12_Br_2_N_2_O_3_
*M* *_r_* = 428.09
Monoclinic, 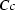

*a* = 10.886 (1) Å
*b* = 12.956 (2) Å
*c* = 10.965 (2) Å
β = 96.476 (3)°
*V* = 1536.6 (4) Å^3^
*Z* = 4
Mo *K*α radiation
μ = 5.29 mm^−1^
*T* = 298 K
0.30 × 0.30 × 0.27 mm

#### Data collection

Bruker SMART CCD diffractometer
Absorption correction: multi-scan (*SADABS*; Bruker, 2001 ▶) *T*_min_ = 0.300, *T*_max_ = 0.329 (expected range = 0.219–0.240)
4623 measured reflections
2208 independent reflections
1992 reflections with *I* > 2σ(*I*)
*R*_int_ = 0.030

#### Refinement

*R*[*F* ^2^ > 2σ(*F* ^2^)] = 0.043
*wR*(*F* ^2^) = 0.125
*S* = 1.05
2208 reflections
204 parameters
3 restraints
H atoms treated by a mixture of independent and constrained refinement
Δρ_max_ = 0.35 e Å^−3^
Δρ_min_ = −0.62 e Å^−3^
Absolute structure: Flack (1983 ▶), 531 Friedel pairs
Flack parameter: 0.513 (19)

### 

Data collection: *SMART* (Bruker, 2007 ▶); cell refinement: *SAINT* (Bruker, 2007 ▶); data reduction: *SAINT*; program(s) used to solve structure: *SHELXTL* (Sheldrick, 2008 ▶); program(s) used to refine structure: *SHELXTL*; molecular graphics: *SHELXTL*; software used to prepare material for publication: *SHELXTL*.

## Supplementary Material

Crystal structure: contains datablocks global, I. DOI: 10.1107/S1600536809022168/hb5004sup1.cif

Structure factors: contains datablocks I. DOI: 10.1107/S1600536809022168/hb5004Isup2.hkl

Additional supplementary materials:  crystallographic information; 3D view; checkCIF report

## Figures and Tables

**Table 1:** Hydrogen-bond geometry (Å, °)

*D*—H⋯*A*	*D*—H	H⋯*A*	*D*⋯*A*	*D*—H⋯*A*
N2—H2⋯O3	0.90 (5)	1.97 (9)	2.617 (8)	128 (9)
O1—H1⋯N1	0.82	1.93	2.535 (7)	130

**Fig. 1.**
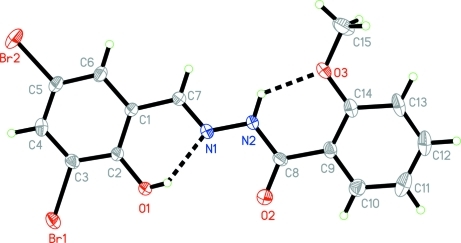
The molecular structure of (I) with ellipsoids drawn at the 30% probability level and hydrogen bonds indicated by dashed lines.

## supplementary crystallographic information

### Comment

Study on the crystal structures of hydrazone derivatives is a hot topic in structural chemistry. In the last few years, the crystal structures of a large number of hydrazone compounds have been reported (Mohd Lair *et al.*, 2009; Fun *et al.*, 2008; Li & Ban, 2009; Zhu *et al.*, 2009; Yang, 2007; You *et al.*, 2008). As a continuation of our work in this area (Qu *et al.*, 2008; Yang *et al.*, 2008), the title new hydrazone compound, (I), derived from the reaction of 3,5-dibromo-2-hydroxybenzaldehyde with an equimolar quantity of 2-methoxybenzohydrazide is reported.

In compound (I), Fig. 1, the dihedral angle between the two benzene rings is 3.4 (2)°. Intramolecular N2—H2···O3 and O1—H1···N1 hydrogen bonds, (Table 1) are observed in the molecule. All the bond lengths are within normal values (Allen *et al.*, 1987).

### Experimental

The title compound was prepared by refluxing equimolar quantities of 3,5-dibromo-2-hydroxybenzaldehyde with 2-methoxybenzohydrazide in methanol. Colorless blocks of (I) were formed by slow evaporation of the solution in air.

### Refinement

Atom H2 was located in a difference Fourier map and refined isotropically, with the N–H distance restrained to 0.90 (1) Å. The other H atoms were placed in idealized positions (C–H = 0.93-0.96 Å, O–H = 0.82 Å) and refined as riding with *U*_iso_(H) = 1.2*U*_eq_(C) or 1.5*U*_eq_(O and methyl C). The crystal studied was an inversion twin with a 0.513 (19):0.487 (19) domain ratio.

### Crystal data

**Table:**

C_15_H_12_Br_2_N_2_O_3_	*F*(000) = 840
*M**_r_* = 428.09	*D*_x_ = 1.850 Mg m^−^^3^
Monoclinic, *C**c*	Mo *K*α radiation, λ = 0.71073 Å
Hall symbol: C -2yc	Cell parameters from 2250 reflections
*a* = 10.886 (1) Å	θ = 2.4–25.9°
*b* = 12.956 (2) Å	µ = 5.29 mm^−^^1^
*c* = 10.965 (2) Å	*T* = 298 K
β = 96.476 (3)°	Block, colorless
*V* = 1536.6 (4) Å^3^	0.30 × 0.30 × 0.27 mm
*Z* = 4	

### Data collection

**Table:**

Bruker SMART CCD diffractometer	2208 independent reflections
Radiation source: fine-focus sealed tube	1992 reflections with *I* > 2σ(*I*)
graphite	*R*_int_ = 0.030
ω scans	θ_max_ = 27.0°, θ_min_ = 2.5°
Absorption correction: multi-scan (*SADABS*; Bruker, 2001)	*h* = −9→13
*T*_min_ = 0.300, *T*_max_ = 0.329	*k* = −16→16
4623 measured reflections	*l* = −13→13

### Refinement

**Table:**

Refinement on *F*^2^	Secondary atom site location: difference Fourier map
Least-squares matrix: full	Hydrogen site location: inferred from neighbouring sites
*R*[*F*^2^ > 2σ(*F*^2^)] = 0.043	H atoms treated by a mixture of independent and constrained refinement
*wR*(*F*^2^) = 0.125	*w* = 1/[σ^2^(*F*_o_^2^) + (0.0877*P*)^2^ + 0.6851*P*] where *P* = (*F*_o_^2^ + 2*F*_c_^2^)/3
*S* = 1.05	(Δ/σ)_max_ = 0.001
2208 reflections	Δρ_max_ = 0.35 e Å^−^^3^
204 parameters	Δρ_min_ = −0.62 e Å^−^^3^
3 restraints	Absolute structure: Flack (1983), 531 Friedel pairs
Primary atom site location: structure-invariant direct methods	Flack parameter: 0.513 (19)

### Special details

**Table:**

Geometry. All esds (except the esd in the dihedral angle between two l.s. planes) are estimated using the full covariance matrix. The cell esds are taken into account individually in the estimation of esds in distances, angles and torsion angles; correlations between esds in cell parameters are only used when they are defined by crystal symmetry. An approximate (isotropic) treatment of cell esds is used for estimating esds involving l.s. planes.
Refinement. Refinement of F^2^ against ALL reflections. The weighted R-factor wR and goodness of fit S are based on F^2^, conventional R-factors R are based on F, with F set to zero for negative F^2^. The threshold expression of F^2^ > 2sigma(F^2^) is used only for calculating R-factors(gt) etc. and is not relevant to the choice of reflections for refinement. R-factors based on F^2^ are statistically about twice as large as those based on F, and R- factors based on ALL data will be even larger.

### Fractional atomic coordinates and isotropic or equivalent isotropic displacement parameters (Å^2^)

**Table:**

	*x*	*y*	*z*	*U*_iso_*/*U*_eq_	
Br1	0.39963 (8)	−0.26669 (6)	−0.15520 (7)	0.0501 (2)	
Br2	0.48191 (8)	−0.46448 (6)	0.30371 (8)	0.0556 (3)	
O1	0.5231 (5)	−0.0896 (4)	−0.0108 (4)	0.0388 (11)	
H1	0.5797	−0.0514	0.0166	0.058*	
O2	0.6295 (7)	0.1701 (5)	0.0431 (5)	0.0548 (16)	
O3	0.7666 (6)	0.2128 (4)	0.4081 (5)	0.0466 (14)	
N1	0.6150 (6)	0.0066 (5)	0.1793 (5)	0.0341 (12)	
N2	0.6578 (6)	0.0982 (4)	0.2312 (5)	0.0360 (12)	
C1	0.5608 (6)	−0.1684 (5)	0.1883 (6)	0.0300 (13)	
C2	0.5188 (6)	−0.1713 (5)	0.0627 (6)	0.0306 (13)	
C3	0.4675 (7)	−0.2629 (5)	0.0147 (7)	0.0351 (14)	
C4	0.4612 (7)	−0.3510 (5)	0.0854 (8)	0.0389 (15)	
H4	0.4296	−0.4122	0.0504	0.047*	
C5	0.5026 (7)	−0.3458 (5)	0.2081 (7)	0.0369 (15)	
C6	0.5533 (7)	−0.2572 (5)	0.2625 (8)	0.0360 (16)	
H6	0.5818	−0.2559	0.3456	0.043*	
C7	0.6101 (7)	−0.0736 (5)	0.2472 (6)	0.0343 (14)	
H7	0.6369	−0.0714	0.3307	0.041*	
C8	0.6631 (7)	0.1797 (6)	0.1527 (6)	0.0332 (14)	
C9	0.7136 (7)	0.2793 (5)	0.2082 (7)	0.0354 (15)	
C10	0.7100 (8)	0.3626 (6)	0.1268 (8)	0.0458 (18)	
H10	0.6764	0.3535	0.0458	0.055*	
C11	0.7548 (10)	0.4567 (7)	0.1642 (12)	0.063 (3)	
H11	0.7503	0.5115	0.1090	0.076*	
C12	0.8069 (9)	0.4715 (7)	0.2835 (11)	0.059 (2)	
H12	0.8380	0.5358	0.3088	0.071*	
C13	0.8126 (8)	0.3906 (7)	0.3647 (9)	0.052 (2)	
H13	0.8486	0.4003	0.4449	0.062*	
C14	0.7652 (7)	0.2940 (6)	0.3286 (7)	0.0386 (15)	
C15	0.8276 (10)	0.2211 (8)	0.5282 (8)	0.062 (3)	
H15A	0.9101	0.2462	0.5248	0.093*	
H15B	0.8309	0.1545	0.5668	0.093*	
H15C	0.7833	0.2682	0.5748	0.093*	
H2	0.692 (9)	0.097 (9)	0.310 (3)	0.080*	

### Atomic displacement parameters (Å^2^)

**Table:**

	*U*^11^	*U*^22^	*U*^33^	*U*^12^	*U*^13^	*U*^23^
Br1	0.0682 (5)	0.0405 (4)	0.0380 (4)	−0.0037 (4)	−0.0105 (3)	−0.0106 (3)
Br2	0.0651 (5)	0.0333 (4)	0.0706 (6)	0.0050 (4)	0.0171 (4)	0.0194 (4)
O1	0.059 (3)	0.029 (2)	0.027 (2)	−0.007 (2)	−0.006 (2)	0.0006 (18)
O2	0.079 (4)	0.043 (3)	0.039 (3)	−0.015 (3)	−0.011 (3)	0.005 (3)
O3	0.060 (4)	0.044 (3)	0.033 (3)	−0.015 (3)	−0.007 (2)	−0.007 (2)
N1	0.042 (3)	0.030 (3)	0.030 (3)	−0.006 (2)	0.001 (2)	−0.005 (2)
N2	0.050 (3)	0.028 (3)	0.029 (3)	−0.010 (3)	−0.003 (2)	−0.003 (2)
C1	0.034 (3)	0.026 (3)	0.030 (3)	−0.001 (2)	0.002 (2)	0.002 (2)
C2	0.036 (3)	0.024 (3)	0.031 (3)	0.002 (2)	0.000 (3)	0.000 (2)
C3	0.036 (4)	0.034 (3)	0.033 (4)	0.003 (3)	−0.004 (3)	−0.003 (3)
C4	0.038 (4)	0.028 (3)	0.050 (4)	0.002 (3)	0.004 (3)	−0.007 (3)
C5	0.040 (4)	0.028 (3)	0.044 (4)	0.005 (3)	0.010 (3)	0.006 (3)
C6	0.039 (4)	0.026 (3)	0.042 (4)	0.000 (3)	0.002 (3)	0.005 (3)
C7	0.044 (4)	0.029 (3)	0.029 (3)	0.001 (3)	−0.001 (3)	−0.001 (2)
C8	0.035 (3)	0.038 (4)	0.025 (3)	−0.004 (3)	0.000 (3)	−0.004 (3)
C9	0.038 (4)	0.032 (4)	0.038 (4)	−0.005 (3)	0.011 (3)	−0.008 (3)
C10	0.051 (5)	0.040 (4)	0.048 (4)	0.000 (3)	0.010 (3)	0.000 (3)
C11	0.063 (6)	0.034 (4)	0.096 (8)	−0.003 (4)	0.026 (6)	0.005 (5)
C12	0.056 (5)	0.036 (4)	0.088 (7)	−0.013 (4)	0.018 (5)	−0.020 (4)
C13	0.046 (4)	0.046 (5)	0.065 (5)	−0.016 (4)	0.012 (4)	−0.030 (4)
C14	0.035 (4)	0.037 (3)	0.045 (4)	−0.007 (3)	0.008 (3)	−0.013 (3)
C15	0.061 (6)	0.086 (7)	0.037 (4)	−0.011 (5)	−0.005 (4)	−0.007 (4)

### Geometric parameters (Å, °)

**Table:**

Br1—C3	1.925 (7)	C5—C6	1.379 (10)
Br2—C5	1.889 (7)	C6—H6	0.9300
O1—C2	1.335 (8)	C7—H7	0.9300
O1—H1	0.8200	C8—C9	1.504 (10)
O2—C8	1.222 (8)	C9—C14	1.388 (11)
O3—C14	1.366 (10)	C9—C10	1.398 (11)
O3—C15	1.411 (11)	C10—C11	1.359 (13)
N1—C7	1.282 (9)	C10—H10	0.9300
N1—N2	1.375 (8)	C11—C12	1.380 (17)
N2—C8	1.367 (9)	C11—H11	0.9300
N2—H2	0.90 (5)	C12—C13	1.372 (14)
C1—C2	1.401 (9)	C12—H12	0.9300
C1—C6	1.417 (9)	C13—C14	1.394 (10)
C1—C7	1.461 (9)	C13—H13	0.9300
C2—C3	1.389 (10)	C15—H15A	0.9600
C3—C4	1.386 (11)	C15—H15B	0.9600
C4—C5	1.372 (11)	C15—H15C	0.9600
C4—H4	0.9300		
			
C2—O1—H1	109.5	O2—C8—N2	120.7 (6)
C14—O3—C15	120.5 (7)	O2—C8—C9	122.7 (6)
C7—N1—N2	119.5 (6)	N2—C8—C9	116.6 (6)
C8—N2—N1	116.3 (5)	C14—C9—C10	118.7 (7)
C8—N2—H2	125 (7)	C14—C9—C8	126.3 (7)
N1—N2—H2	118 (7)	C10—C9—C8	114.9 (7)
C2—C1—C6	120.5 (6)	C11—C10—C9	121.1 (9)
C2—C1—C7	121.3 (6)	C11—C10—H10	119.4
C6—C1—C7	118.1 (6)	C9—C10—H10	119.4
O1—C2—C3	119.2 (6)	C10—C11—C12	120.3 (9)
O1—C2—C1	122.8 (6)	C10—C11—H11	119.8
C3—C2—C1	117.9 (6)	C12—C11—H11	119.8
C4—C3—C2	122.3 (7)	C13—C12—C11	119.6 (8)
C4—C3—Br1	118.8 (5)	C13—C12—H12	120.2
C2—C3—Br1	118.9 (5)	C11—C12—H12	120.2
C5—C4—C3	118.5 (6)	C12—C13—C14	120.9 (9)
C5—C4—H4	120.8	C12—C13—H13	119.6
C3—C4—H4	120.8	C14—C13—H13	119.6
C4—C5—C6	122.4 (6)	O3—C14—C9	118.4 (6)
C4—C5—Br2	117.3 (5)	O3—C14—C13	122.2 (7)
C6—C5—Br2	120.3 (6)	C9—C14—C13	119.4 (8)
C5—C6—C1	118.3 (7)	O3—C15—H15A	109.5
C5—C6—H6	120.9	O3—C15—H15B	109.5
C1—C6—H6	120.9	H15A—C15—H15B	109.5
N1—C7—C1	117.5 (6)	O3—C15—H15C	109.5
N1—C7—H7	121.3	H15A—C15—H15C	109.5
C1—C7—H7	121.3	H15B—C15—H15C	109.5

### Hydrogen-bond geometry (Å, °)

**Table:**

*D*—H···*A*	*D*—H	H···*A*	*D*···*A*	*D*—H···*A*
N2—H2···O3	0.90 (5)	1.97 (9)	2.617 (8)	128 (9)
O1—H1···N1	0.82	1.93	2.535 (7)	130
